# Epigenetic editing of the *STAT5B* promoter attenuates milk nutrient loss in a bovine mastitis cell model

**DOI:** 10.1093/procel/pwaf113

**Published:** 2026-01-02

**Authors:** Sixue Li, Xiao Li, Qing Liu, Yongwang Miao, Le Kang, Feng Jiang

**Affiliations:** State Key Laboratory of Animal Biodiversity Conservation and Integrated Pest Management, Institute of Zoology, Chinese Academy of Sciences, Beijing 100101, China; College of Life Sciences, University of Chinese Academy of Sciences, Beijing 100101, China; State Key Laboratory of Animal Biodiversity Conservation and Integrated Pest Management, Institute of Zoology, Chinese Academy of Sciences, Beijing 100101, China; Guangzhou National Laboratory, Guangzhou 510300, China; State Key Laboratory of Animal Biodiversity Conservation and Integrated Pest Management, Institute of Zoology, Chinese Academy of Sciences, Beijing 100101, China; Faculty of Animal Science and Technology, Yunnan Agricultural University, Kunming 650201, China; State Key Laboratory of Animal Biodiversity Conservation and Integrated Pest Management, Institute of Zoology, Chinese Academy of Sciences, Beijing 100101, China; College of Life Sciences, University of Chinese Academy of Sciences, Beijing 100101, China; State Key Laboratory of Animal Biodiversity Conservation and Integrated Pest Management, Institute of Zoology, Chinese Academy of Sciences, Beijing 100101, China; College of Life Sciences, University of Chinese Academy of Sciences, Beijing 100101, China

**Dear Editor,** 

Mastitis is one of the most economically impactful diseases during the lactation cycle of modern high-yielding dairy cows (Aghamohammadi et al., 2018). Contagious bacterial infections are central to its pathogenesis, among which *Staphylococcus aureus* is the most prevalent causative agent (Bradley, 2002). Of particular clinical significance, *S. aureus* predominantly causes persistent subclinical mastitis, which exhibits resistance to conventional therapeutic interventions (Eckel and Ametaj, 2016; Strandberg et al., 2005). Subclinical mastitis induced by *S. aureus* not only increases production costs due to treatment and milk discard but also reduces the commercial value of products, highlighting the urgent need for effective control measures (Halasa et al., 2007). Current treatments for bovine mastitis rely mainly on antibiotics and vaccination. However, the efficacy of antibiotics is increasingly constrained by rising resistance, while vaccine protection is often insufficient due to the polymicrobial nature of the disease (Ashraf and Imran, 2020). Consequently, novel strategies remain necessary to combat subclinical mastitis induced by *S. aureus*.

Subclinical mastitis induced by *S. aureus* infection disrupts the expression of lactation-related genes, leading to irreversible reductions in milk yield and quality. Consequently, even after clinical resolution of the infection, affected dairy cows often fail to regain their peak production levels throughout the remainder of lactation cycle (Hertl et al., 2014). The inflammatory response triggered by *S. aureus* components such as peptidoglycan (PGN) and lipoteichoic acid (LTA) disrupts mammary function, impairing synthesis of milk proteins and fats (Wu et al., 2020a). The key pathways including JAK2-STAT5 are suppressed, reducing expression of critical genes like *CSN2* (Wu et al., 2020b). Epigenetic mechanisms such as HDAC-mediated deacetylation further contribute to the repression of lactogenic genes (Wu et al., 2020a). Given that the impaired protein synthesis and nutritional losses in subclinical mastitis result from the downregulation of lactogenic gene expression, restoring the expression of these lactogenic genes theoretically provides a pathogen-tailored strategy to preserve milk quality.

Lactation in mammals is regulated by prolactin (PRL) in a concentration-dependent manner. Treatment of MAC-T cells (an immortalized bovine mammary cell line) with 10 ng/mL PRL (Ma et al., 2024) induced a range of lactogenic responses, including increased lipid droplet accumulation (Fig. 1A and 1B, *P *< 0.001), elevated levels of casein and triglycerides (Fig. 1C, *P *< 0.001), and the upregulation of most lactogenic genes (*mTOR*, *PER2*, *PPrPγ*, *FASN*, *SREBF1*, *STAT5A*, *STAT5B*, *JAK2*) except *AKT1* and *CSN2* (Fig. S1—see online supplementary material for a colour version of this figure, *P *< 0.05). Consistent with these findings, western blot analysis confirmed elevated protein levels of FASN, STAT5A, AKT1, SCD, and CSN2 after 72 h (Fig. S2—see online supplementary material for a colour version of this figure, *P *< 0.05). To identify the optimal PRL concentration for inducing differentiation in bovine mammary epithelial cells (BMECs), we treated cells with a range of PRL doses and evaluated lipid droplet accumulation via BODIPY and Oil Red O staining. Low and high PRL concentrations led to suboptimal differentiation, while 50 ng/mL was identified as the most effective dose for lipid droplet accumulation (Figs. S3 and S4—see online supplementary material for a colour version of these figures, *P *< 0.001). Compared with the prolactin-free group, this concentration significantly increased triglyceride levels but did not affect casein levels (Figs. S5 and S6—see online supplementary material for a colour version of these figures; *P *< 0.01 for triglycerides and *P *> 0.05 for casein). This concentration also significantly upregulated the mRNA levels of key genes involved in lipid synthesis (*FASN*, *ACACA*, *SCD*) and protein synthesis (*STAT5B*, *CSN2*), relative to the prolactin-free group (*P *< 0.05, Fig. S7—see online supplementary material for a colour version of this figure). Thus, PRL and other hormones effectively induce lactogenic differentiation in bovine mammary epithelial cells.

**Figure 1.**
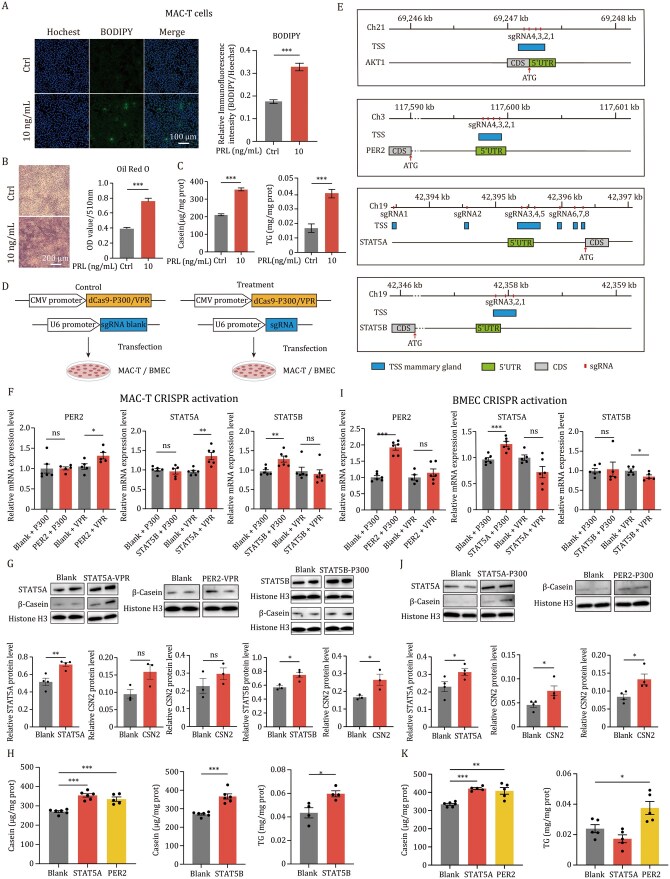
CRISPR/dCas9-based epigenetic editing activates lactation genes to increase milk protein and milk fat. (A) Lipid droplets fluorescence staining and quantification of MAC-T cells before and after differentiation (*n *= 4). Left: BODIPY stains the lipid droplets, and Hoechst stains the cell nuclei; Right: The relative fluorescence intensity of lipid droplets as calculated by ImageJ (BODIPY/Hoechst). Blue, Hoechst; Green, BODIPY. Scale bar, 100 μm. (B) Oil Red O staining and quantification of MAC-T cells before and after differentiation (*n *= 4). Left: Oil Red O stains the lipid droplets, and Hematoxylin stains the cell nuclei; Right: Quantitative analysis of 510 nm absorbance after oil red staining. Dark blue, Hematoxylin; Red, Oil Red O. Scale bar, 200 μm. (C) Casein and triglyceride (TG) contents in MAC-T cells before and after differentiation (*n *= 6). (D) The schematic diagram of CRISPR activation design. (E) The schematic diagram of transcription initiation site (TSS) and sgRNAs of *AKT1*, *PER2*, *STAT5A*, and *STAT5B*. (F) q-PCR results of the CRISPR activation testing of four genes (*AKT1*, *PER2*, *STAT5A*, *STAT5B*) in MAC-T cells (*n *= 5). The meaning of *x*-axis: Blank + P300 = non-targeting sgRNA + dCas9-P300; STAT5A + P300 = dCas9-P300 targeting STAT5A. (G) Western blot analysis of STAT5A and CSN2 proteins after *STAT5A* CRISPRa, CSN2 proteins after *PER2* CRISPRa, and STAT5B and CSN2 proteins after *STAT5B* CRISPRa in MAC-T cells, Histone H3 was used as a loading control (*n *= 4, 3). (H) Casein and TG content in MAC-T cells after the activation of *STAT5A, PER2*, and *STAT5B* (*n *= 6, 4). (I) q-PCR results of the CRISPR activation screening of four genes (*AKT1*, *PER2*, *STAT5A*, *STAT5B*) in BMECs. Non-targeting sgRNA was used as a control (*n *= 5). (J) Western blot analysis of STAT5A and CSN2 proteins after *STAT5A* CRISPRa, CSN2 proteins after *PER2* CRISPRa in BMECs. Histone H3 was used as a loading control (*n *= 4). (K) Casein and TG content of activating *STAT5A* and *PER2* in BMECs (*n *= 5). Values are means and error bars indicate SEM. *P < 0.05; **P < 0.01; ***P < 0.001. Data were analyzed by Student’s *t*-tests.

To enhance nutritional components, we overexpressed the key lactogenic genes (*AKT1*, *PER2*, *STAT5A*, *STAT5B*) via plasmid transfection. The qPCR analysis confirmed the successful upregulation of their expression in both MAC-T cells and BMECs (Figs. S8 and S9—see online supplementary material for a colour version of these figures, *P *< 0.0001). In MAC-T cells, the overexpression of *AKT1*, *STAT5A*, and *STAT5B* increased casein, while the overexpression of *AKT1*, *PER2*, and *STAT5A* resulted in the elevated TG levels (Figs. S10 and S11—see online supplementary material for a colour version of these figures, *P *< 0.05). In BMECs, the overexpression of *AKT1* and *STAT5A* raised the casein levels, while the overexpression of *STAT5B* increased the TG levels (Figs. S12 and S13—see online supplementary material for a colour version of these figures, *P *< 0.05). These results indicate cell type-specific effects on nutrient synthesis.

Next, we aimed to determine whether the overexpression using epigenetic editing can recapitulate the effect of plasmid-based overexpression via the coding region in enhancing nutrient production. To induce gene activation, we utilized two epigenetic regulators, the transcriptional activator dCas9-VPR and the chromatin remodeler dCas9-P300, while an sgRNA backbone plasmid acted as the negative control (Fig. 1D). We identified the promoter regions of *AKT1*, *PER2*, *STAT5A* and *STAT5B* based on transcriptional start site annotation from the FAANG Genome Browser. To enable comprehensive coverage of chromatin-accessible regions proximal to the transcription start site (TSS), we designed multiple sgRNAs for each target gene (Fig. 1E). In MAC-T cells, dCas9-VPR activated the expression of *PER2* and *STAT5A* (*P *< 0.05, Student’s *t*-test), while dCas9-p300 activated the *STAT5B* expression (Fig. 1F, *P *< 0.01, Student’s *t*-test). The overexpression of *STAT5A* and *STAT5B* significantly increased their protein levels compared to the vector controls (Fig. 1G, *P *< 0.05, Student’s *t*-test), whereas a significant increase in CSN2 protein levels was detected only for *STAT5B*, but not for *STAT5A* (*P *< 0.05, Student’s *t*-test). Due to the unavailability of a commercial PER2 antibody, we only quantified the protein level of CSN2 and found no significant difference in CSN2 protein levels after *PER2* activation (Fig. 1G, *P *= 0.27, Student’s *t*-test). The functional analyses demonstrated that the activation of all three target genes (*PER2*, *STAT5A*, and *STAT5B*) significantly increased the β-casein levels (Fig. 1H, *P *< 0.001, Student’s *t*-test), but only the *STAT5B* activation produced a statistically significant increase in TG levels (Fig. 1H, *P *< 0.05, Student’s *t*-test). Conversely, in BMECs, the expression of *PER2* and *STAT5A* was significantly upregulated by dCas9-P300, but not by dCas9-VPR (Fig. 1I, *P *< 0.001, Student’s *t*-test). Furthermore, the overexpression of *STAT5A* and *PER2* led to the increased protein expression of themselves and the elevated CSN2 protein levels in BMECs (Fig. 1J, *P *< 0.05, Student’s *t*-test). Consistently, the overexpression of *PER2* and *STAT5A* significantly enhanced the casein production (Fig. 1K, *P *< 0.01, Student’s *t*-test), while only *PER2* activation increased the TG levels (Fig. 1K, *P *< 0.05, Student’s *t*-test). The *AKT1* gene was not activated in either of the two types of cells (Fig. S14—see online supplementary material for a colour version of this figure, *P *> 0.05, Student’s *t*-test). Finally, we evaluated the activation efficiency of individual sgRNAs from successfully activated mixtures (Figs. S15–19—see online supplementary material for a colour version of these figures). We found that the expression of *STAT5A* and *PER2* was significantly upregulated by dCas9-VPR and dCas9-P300 using a single gRNA in both MAC-T cells and BMECs (*P *< 0.05, Student’s *t*-test). However, the expression of *STAT5B* could not be activated by dCas9-P300 using any single gRNA in MAC-T cells, suggesting a synergistic effect of multiple gRNAs. These results demonstrate that epigenetic editing can activate the expression of lactation genes, thereby enhancing the synthesis of nutritional components in bovine mammary epithelial cells.

To determine whether epigenetic editing can be used to restore nutritional deficits in mastitis induced by *S. aureus* infection, we developed an *in vitro* mastitis model by exposing differentiated MAC-T cells and BMECs to heat-inactivated *S. aureus* at a 1:10 (cell: bacteria) ratio for 3 h and 24 h. The morphological examination showed progressive cellular damage and increased debris accumulation compared to untreated controls (Fig. 2A, *P *< 0.001, one-way ANOVA). The cell viability assays revealed time-dependent reductions. Specifically, MAC-T cells exhibited the significant viability decreases as early as 3 h (Fig. 2B, *P *< 0.01), whereas BMECs showed the significant viability loss only at 24 h (Fig. 2C, *P *< 0.01, one-way ANOVA). Since bovine mastitis response is characterized by dramatic upregulation of key cytokines (*IL-6*, *IL-8*, and *IL-1β*) (Ran et al., 2020), we quantified their expression at 3 h and 24 h post-infection in MAC-T cells. We found that their expression reached peak levels at 3 h, consistent with the characteristics of bovine mastitis (Fig. 2D and 2E, *P *< 0.0001, Student’s *t*-test). Only the MAC-T cells, but not the BMECs, exhibited a statistically significant suppression of casein levels at 24 h post-induction (Fig. 2F and 2G, *P *< 0.01, one-way ANOVA). However, the TG levels remained unchanged in both cell lines (Fig. 2F and 2G, *P > *0.05, one-way ANOVA). In addition, MAC-T cells displayed a marked reduction of *STAT5B* expression at both 3 h and 24 h post-infection, whereas a significant reduction in *STAT5A* expression was observed only at 3 h post-infection (Fig. 2H, *P *< 0.0001, one-way ANOVA). In contrast, the expression of *PER2*, *STAT5A*, and *STAT5B* showed no significant changes in BMECs (Fig. 2I, *P *< 0.0001, one-way ANOVA). Together, these results confirmed the successful establishment of mastitis models in MAC-T cells that recapitulate the characteristic mastitis response, including suppression of *STAT5A* and *STAT5B* expression and the nutritional impairment observed in bovine mastitis.

**Figure 2.**
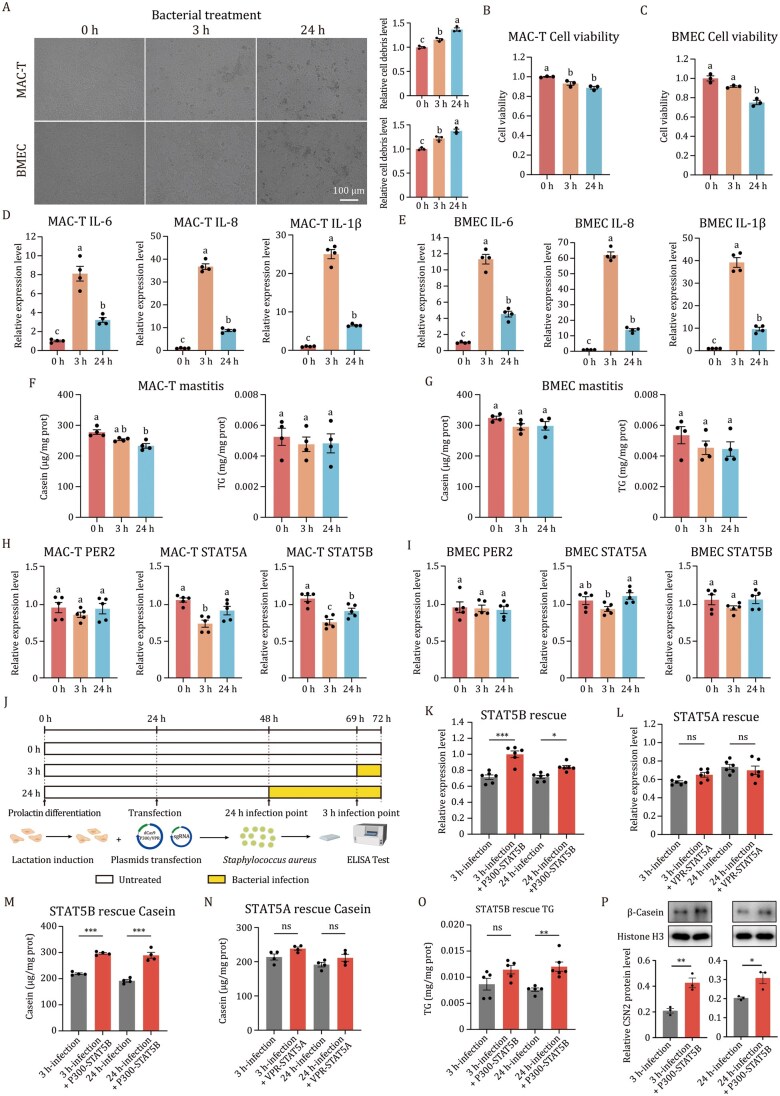
STAT5B epigenetic activation restores casein and TG levels in the mastitis cell model. (A) Phase-contrast microscopy at different time points in bovine mastitis induced by *Staphylococcus aureus* and calculate the proportion of cell debris on the grayscale image using ImageJ (*n *= 3). Scale bar, 100 μm. (B) CCK-8 cell viability assays of MAC-T cells under mastitis conditions (*n* = 3). (C) The CCK-8 cell viability results of mastitis BMECs (*n *= 3). (D) q-PCR analysis of *IL-6*, *IL-8* and *IL1-β* in mastitis MAC-T cells. *β-actin* was used as a control (*n *= 4). (E) q-PCR analysis of *IL-6*, *IL-8* and *IL1-β* in mastitis BMECs, *β-actin* served as control (*n *= 4). (F) Casein and TG content of mastitis MAC-T cells (*n *= 4). (G) Casein and TG content of mastitis BMECs (*n *= 4). (H) q-PCR analysis of *PER2*, *STAT5A* and *STAT5B* in mastitis MAC-T cells (*n *= 5). (I) q-PCR analysis of *PER2*, *STAT5A* and *STAT5B* in mastitis BMECs (*n *= 5). (J) Schematic diagram of the epigenetic activation experiment of the mastitis cell model. MAC-T cells were transfected with dCas9 effector and, 24 h later, challenged with 5 × 10^7^/mL heat-inactivated *S. aureus* for 3 h or 24 h. Unchallenged cells were cultivated in parallel as controls. (K) q-PCR analysis of *STAT5B* after epigenetic activation in mastitis cells (*n *= 6). (L) q-PCR analysis of *STAT5A* after epigenetic activation in mastitis cells (*n *= 6). (M) Casein content in mastitis MAC-T cells after CRISPR activation of *STAT5B* (*n *= 4). (N) Casein content in mastitis MAC-T cells after CRISPR activation of *STAT5A* (*n *= 4). (O) TG content in mastitis MAC-T cells after CRISPR activation of *STAT5B* (*n *= 5). (P) CSN2 protein levels in 3 h or 24 h-infection mastitis MAC-T cells after CRISPR activation of *STAT5B* (*n *= 3). Values are means and error bars indicate SEM. *P < 0.05; **P < 0.01; ***P < 0.001. Data were analyzed by ordinary one-way ANOVA tests for (B–I) and by Student’s *t*-test for (K–P), respectively.

To evaluate the efficacy of epigenetic editing in restoring nutritional components in the mastitis model, the MAC-T cells were seeded and cultured to 80% confluence before transfection with the dCas9-VPR/P300 epigenetic editing system containing a mixture of sgRNAs separately targeting *STAT5A* and *STAT5B* (Fig. 2J). The q-PCR analysis showed that dCas9-P300 significantly activated the *STAT5B* expression at both 3 h and 24 h post-infection (Fig. 2K, *P *< 0.05, Student’s *t*-test), whereas the *STAT5A* expression was not significantly changed by dCas9-VPR at these two time points (Fig. 2L, *P *> 0.05, Student’s *t*-test). Accordingly, a significant increase in casein levels was observed for dCas9-P300-STAT5B (Fig. 2M, *P *< 0.001, Student’s *t*-test), but not for dCas9-VPR-STAT5A, at both 3 h and 24 h post-infection (Fig. 2N, *P *> 0.05, Student’s *t*-test). Furthermore, the levels of TG (Fig. 2O, *P *< 0.01, Student’s *t*-test) and CSN2 protein (Fig. 2P, *P *< 0.05, Student’s *t*-test) were significantly increased by dCas9-P300-STAT5B. Taken together, these results showed that the restoration of *STAT5B* expression through epigenetic editing rescued the impairment of nutritional components caused by *S. aureus* infection in the mastitis model established in MAC-T cells.

In this study, we established a link between lactogenic gene expression and cellular differentiation in bovine mammary epithelial cells. We found that overexpression of lactation genes via plasmid transfection and epigenetic editing enhanced milk nutrient synthesis. Under mastitis conditions induced by *S. aureus*, epigenetic activation of *STAT5B* but not *STAT5A* rescued casein production, highlighting its role as a master regulator of lactation (Liu et al., 1996). Notably, combinatorial sgRNA design synergistically enhanced *STAT5B* expression, suggesting strong cooperative interaction among sgRNAs (Doench et al., 2016). This finding underscores the importance of optimizing multi-sgRNA strategies for efficient epigenetic editing and suggests that spatial organization of target sites may critically influence editor efficacy (Note S1—see online supplementary material). Additionally, the greatest restoration of nutritional components was achieved through *STAT5B* activation, highlighting the importance of prioritizing core regulatory factors in the design of epigenetic interventions (Note S2—see online supplementary material). Conversely, the failure to activate *STAT5A* is likely attributed to differences in the efficiency of the epigenetic editors (dCas9-P300 for STAT5B and dCas9-VPR for STAT5A) due to distinct chromatin contexts (Note S3—see online supplementary material) (Wu et al., 2023). We acknowledge that our mastitis model using heat-inactivated *S. aureus* has inherent limitations, including the lack of virulence factors, oversimplified host-pathogen interactions, and the induction of an acute inflammatory response that does not fully replicate the persistent nature of subclinical mastitis (Note S4—see online supplementary material) (Tsugami et al., 2021). Secondly, and equally important, the persistence of dCas9-P300/VPR-mediated activation and the direct evidence for chromatin remodeling (e.g., as measurable by ChIP-seq or ATAC-seq) remain uncharacterized. Addressing these aspects is a crucial objective for future research before any potential application. Nevertheless, our study provides a proof-of-concept for lactation-focused interventions and offers a foundation for future studies in more physiologically relevant models. As summarized in the schematic model (Fig. S21—see online supplementary material for a colour version of this figure), our study demonstrates that targeted epigenetic editing of lactogenic genes such as STAT5B enhances milk nutrient synthesis under physiological conditions. More importantly, in the context of the *S. aureus*-induced mastitis model, targeted *STAT5B* promoter editing via dCas9-P300 effectively reverses the infection-driven nutritional impairment, validating its role in restoring lactation function.

## Supplementary Material

pwaf113_Supplementary_Data

## Footnotes

We thank to Wenjing Fang for help with RNA isolation and qPCR experiments. This work was supported by the Agricultural Science and Technology Major Project and the Biological Breeding-National Science and Technology Major Project (2024ZD04077). The funders had no role in the study design, data collection, analysis, decision to publish, or preparation of the manuscript.

The authors declare no competing interests. All authors agree to publish.

F.J., L.K., and Y.M. designed and supervised the study. S.L. performed the experiments. S.L., X.L., Q.L., and F.J. analyzed the data and discussed the results. S.L., Y.M., L.K. and F.J. wrote the paper with input from all authors.
