# Supplementary material for: Epigenetic editing of the STAT5B promoter attenuates milk nutrient loss in a bovine mastitis cell model
Source: Protein Cell. 2026 Jan 2;17(6):578–83. doi: 10.1093/procel/pwaf113 (PMC13225728; doi:10.1093/procel/pwaf113)
Supplement: pwaf113_Supplementary_Data [file pwaf113_supplementary_data.zip › pwaf113_Supplementary_Data/epigeneticEditingBovine.supply.260107.docx]

SUPPLEMENTARY MATERIAL

Epigenetic editing of the *STAT5B* promoter attenuates milk nutrient loss in a bovine mastitis cell model

Sixue Li^1, 2^, Xiao Li^1,3^, Qing Liu^1^, Yongwang Miao^4 #^, Le Kang^1, 2 #^, Feng Jiang^1, 2 #^

^1^ State Key Laboratory of Animal Biodiversity Conservation and Integrated Pest Management, Institute of Zoology, Chinese Academy of Sciences, Beijing, China

^2^ College of Life Sciences, University of Chinese Academy of Sciences, Beijing, China

^3^ Guangzhou National Laboratory, Guangzhou, China

^4^ Faculty of Animal Science and Technology, Yunnan Agricultural University, Kunming, Yunnan, China

**^#^**Corresponding author:

Le Kang, Ph.D

CAS Distinguished Professor

Institute of Zoology, Chinese Academy of Sciences (CAS)

Beijing 100101, China

Tel: 86-10-6480-7219

Fax: 86-10-6480-7099

E-mail: lkang@ioz.ac.cn

or

Yongwang Miao, Professor, Yunnan Agricultural University, miaoyw1@ynau.edu.cn

or

Feng Jiang, Professor, Institute of Zoology, Chinese Academy of Sciences, jiangf@ioz.ac.acn

# MATERIALS AND METHODS

**Cell culture**

Bovine mammary epithelial cells (BMECs) (WHELAB [C3058] , Shanghai, China) and MAC-T cells (WHELAB [C3157]) were provided by Shanghai WHELAB Bioscience Limited. BMECs were cultured in Dulbecco’s modified Eagle medium (DMEM) (Invitrogen, CA, USA) supplemented with 10% fetal bovine serum (FBS) (Royacel, Lanzhou, China) at 37℃ under 5% CO₂. Lactogenic differentiation medium was prepared by supplementing growth medium with 5 μg/mL insulin (MCE, New Jersey, USA), 5 μg/mL hydrocortisone (MCE, New Jersey, USA), 10 or 50 ng/mL prolactin (MCE, New Jersey, USA), and 40 ng/mL EGF (MCE, New Jersey, USA). Cells were passaged or used for experiments at 80% confluency.

**Plasmid construction**

DNA sequences and primers were designed based on the *Bos tauru* reference genome (ARS-UCD2.0, GenBank accession: GCF_002263795.3). Overexpression constructs were synthesized and cloned into the BamHI site of pcDNA3.1(+) by Tianyi Huiyuan (Beijing). The dCas9-P300 vector was purchased from Youbio Biological Technology, and dCas9-VPR-GFP was obtained from Hunan Fenghui Biotechnology. The h(U6)-sgRNA-BFP backbone was gifted by Dr. Haoyi Wang (IOZ, CAS). The mCherry was constructed in dCas9-P300 plasmid by In-Fusion Snap Assembly Master Mix (TAKARA, Beijing). sgRNAs targeting transcriptional start sites (TSS) of candidate genes (FAANG database: https://data.faang.org/genome_browser) were designed and cloned into sgRNA-backbone following the Zhang lab protocol (Ran et al., 2013). The detail of the sgRNAs were listed in the **Appendix Table S1**.

**Lipid droplet staining**

BODIPY (MCE, New Jersey, USA) and Nile Red (MCE, New Jersey, USA) dyes were employed to stain lipid droplets, respectively. Briefly, cells were washed twice with PBS, followed by incubating with 5 μM BODIPY solution for 30 min in darkness. Cell nuclei were stained with 5 μM Hoechst 33342 (MCE, New Jersey, USA) for 10 min. And then washed cells as before. Observation is carried out using fluorescence microscopy.

**Cell transfection**

The activate functionality of sgRNAs was tested by transiently co-transfecting a mix of dCas9-VPR-cherry vector and sgRNA-BFP vector into BMECs and MAC-T cells. For transfection experiments, 2×10^5^ cells were plated into 12-well plates one day before transfection. In total, 1 ug of plasmids mix was transfected using lipofectamine 3000 (Invitrogen, CA, USA) every well according to the instructions. The pooled sgRNA of each gene were using an equimolar mix. The ratio of sgRNA vector mixture: dCas9 vector = 1: 2 was used. The effect of sgRNA activation was represented as fold change relative to the control sgRNAs group.

**Oil Red O staining**

Oil Red O(ORO) staining was measured using an Oil Red O Stain Kit (Solarbio, Beijing, China) according to the manufacturer’s instructions. First, remove the cell culture medium, wash it twice with PBS, and fix it with ORO Fixative for 30 min. Then discard the ORO Fixative and wash it twice with ddH2O. Add 60% isopropanol and soak for 20 s. After discarding 60% isopropanol, soak in newly prepared ORO Stain for 20 min. After that, discard the staining solution and rinse with 60% isopropanol for 20 s. Wash with water 3 times until there is no excess dye. Add Mayer Hematoxylin Solution and counterstain the nucleus for 2 min. After discarding the dye, wash it as before. Add Oil Red O buffer for 1min and discard it. Images were then acquired using an Olympus TH4-200 microscope. Finally, quantitative analysis was carried out by extracting ORO dye with 100% isopropanol and measuring the absorbance value at 510 nm (OD).

**Establishment of cell mastitis model**

Mastitis was induced using *Staphylococcus aureus* (ATCC 6538) cultured in Lysogeny Broth (LB) medium at 37°C with shaking (180 rpm) until mid-logarithmic growth phase (OD600 = 0.5, corresponding to ~5 × 10^7^/mL as enumerated by hemocytometer counting). Heat-killed S. aureus (80°C, 1 h) was pelleted (3,000 × g, 15 min), washed twice in serum-free DMEM, and resuspended in 10% FBS-supplemented DMEM at 1 × 10^9^/mL for cell treatments. 48 h after seeding, the cells reached ~ 90% confluency, bovine mammary epithelial cells were challenged with heat-inactivated *S. aureus* (Final concentration is 1×10^8^/mL) for defined durations (0, 3, 24 h) (Jaeger et al., 2015; Liu et al., 2024; Sajjanar et al., 2019). Following treatment, cells were harvested for downstream analysis.

**RNA Extraction and qRT-PCR**

Total RNA was extracted from cells by using TRIzol (Invitrogen, CA, USA). The concentration of RNA was determined by NanoDrop One (ThermoFisher Scientific, MA, USA). The cDNA of mRNA was synthesized using FastKing RT Kit (Tiangen, Beijing, China). Q-PCR was performed using SYBR Green for cDNA in a LightCycler 480 instrument. All primers used are shown in **Appendix Table S2**.

**Bovine Casein (CSN) ELISA assay**

The cellular Bovine Casein content in BMECs was quantified with the Bovine CSN ELISA Kit (mlbio, Shanghai, China) following the manufacturer’s instructions. In detail, add 50 μl of standard or sample to the 96-well plates. Then add 100 μl of enzyme conjugate to standard wells and sample wells except the blank well, cover with an adhesive strip and incubate for 1 h at 37°C. After that, wash the plate 5 times and append substrate A 50 μl and substrate B 50 μl to each well. Gently mix and incubate in the darkness for 15 minutes at 37°C. Finally, absorbance at 450 nm was measured immediately after adding the stop solution. The Bovine CSN content in samples was determined by calculating against a standard curve according to the manufacturer’s protocols. The relative content of casein was corrected by intracellular protein levels and displayed as micrograms per milligram of protein (µg/mg protein), for which the protein content was determined via pierce BCA Protein Assay Kit (Thermo Fisher Scientific, MA, USA)

**Triglyceride (TG) content assay**

Triglyceride Content Assay Kit (Solarbio, Beijing, China) was used to detect the TG from the BMECs after treatment. TG was extracted from the cells using Reagent I as per the instructions. Subsequently, Reagents II, III, IV, V and Ⅵ were added sequentially, followed by incubation in a water bath at 65 °C. After cooling, absorbance at 420 nm was measured. Using the standard triglyceride as the control, calculate the TG content of each sample based on the obtained OD values. The relative content of triacylglycerol was corrected by intracellular protein levels and displayed as milligrams per milligram of protein (mg/mg protein).

**Protein extract and Western blot**

To extract total proteins from cells, RIPA Lysis Buffer (Proteintech, Wuhan, China) containing PhosSTOPTM and cOmpleteTM (Roche, Basel, Switzerland) was used to lyse cells. The protein concentration was quantified using the Micro BCA Protein Assay Kit (Thermo scientific, MA, USA). The prepared samples were separated on 10% SDS‐polyacrylamide gel and transferred to a PVDF membranes (Millipore, Massachusetts, USA) after cropping according to marker positions. The membranes were incubated in TBST buffer containing 5% skim milk for 1 h at 37℃, followed by incubation in diluted primary antibodies: anti‐AKT1 (1:1000; A5523; ABclonal), anti‐Histone H3 (1:5000; BE3015; Bioeasy), anti‐CSN2 (1:750; A12749; ABclonal), and anti‐FASN (1:1000; AP19050; ABclonal), anti‐SCD (1:1000; A25168; ABclonal), anti‐STAT5A (1:1000; A7733; ABclonal), anti‐STAT5B (1:1000; A12356; ABclonal) overnight at 4℃. Histone H3 was used as an endogenous control. Goat anti-mouse/rabbit IgG was used as the secondary antibody (1:5000, BE0101-100, Bioeasy). Immunoreactive proteins were detected using the Enhanced Chemiluminescence Detection System, and the results were analyzed using ImageJ.

**Statistical analysis**

Data analysis and visualization were carried out using the GraphPad Prism (version 8.0.2) software. All data were presented as the mean ± standard error of the mean. An unpaired two-tailed t-test was employed to compare the two groups, while a one-way ANOVA was used for multiple comparisons, followed by Tukey’s multiple comparisons test. Differences were considered statistically significant at *P*-value < 0.05 (*, **, and *** represent *P* < 0.05, *P* < 0.01, and *P* < 0.001, respectively).

# SUPPLEMENTARY TEXT

**Supplementary Note 1: Combinatorial sgRNA design synergistically enhances gene expression activation**

We evaluated the performance of multiple sgRNAs designed for the epigenetic activators dCas9-P300 and dCas9-VPR in BMECs and MAC-T cells. The expression activation outcomes varied substantially across different sgRNAs. Some sgRNAs inducing moderate upregulation, whereas others resulted in negligible or even repressive effects, indicating that the efficacy of epigenetic editing is strongly influenced by sgRNA selection. Notably, the STAT5B expression activation exhibited a unique dependence on combinatorial sgRNA effects. Although none of the individual sgRNAs targeting *STAT5B* achieved significant gene expression activation in MAC-T cells, the mixed transfection of multiple sgRNAs resulted in robust transcriptional induction, suggesting strong synergistic interaction among sgRNAs (Doench et al., 2016). This synergistic effect implies that multiple targeting of promoters may facilitate enhanced recruitment of epigenetic modifiers, promote local chromatin opening, or stabilize the binding of transcriptional complexes. It underscores the importance of optimizing multi-sgRNA strategies for efficient epigenetic editing and suggests that spatial organization of target sites may critically influence editor efficacy. To further investigate this mechanism, further studies employing ChIP-seq or ATAC-seq can determine whether synergistic expression activation is associated with the changes of histone acetylation or chromatin accessibility at the *STAT5B* locus. These findings emphasize that sgRNA efficacy is not solely determined by individual guide activity but is also shaped by cooperative interactions that emerge in a context-specific manner.

**Supplementary Note 2: Targeting upstream master regulators enables effective epigenetic intervention**

In this study, *STAT5B* was selected as the primary target for epigenetic activation due to its pivotal function as a transcriptional amplifier within prolactin-JAK-STAT signaling cascade. *STAT5B* activation initiates a broad rewiring of lactogenic gene networks, promoting homodimerization or heterodimerization with *STAT5A* and subsequent binding to gamma-interferon activated sites in the promoters of key milk synthesis genes, including *CSN2* (Liu et al., 1996). This upstream positioning allows *STAT5B* to exert pleiotropic effects on downstream processes including protein synthesis, lipid metabolism, and secretory differentiation. Under mastitis conditions, in which multiple lactogenic pathways are suppressed, *STAT5B* expression activation is particularly effective due to its ability to bypass inhibitory signals and directly reinstate transcriptional programs essential for milk production. In contrast, *STAT5A* may serve more context-specific or compensatory roles, while *AKT1* primarily influences parallel metabolic pathways that do not fully overlap with casein regulation. The superior performance of *STAT5B* editors highlights the value of targeting master regulators that occupy central positions in regulatory networks, especially when seeking to reverse complex functional deficits in disease models. This approach offers broader transcriptional leverage and greater functional recovery compared to targeting downstream or pathway-specific genes. Although recent studies, such as *AKT1* promoter demethylation via dCas9-Tet1, have shown partial recovery of CSN2 protein level (Liu et al., 2024), our system achieved more substantial functional restoration, likely owing to direct transcriptional activation of a central lactogenic regulator. Therefore, because targeting upstream master regulators provides maximal leverage for restoring complex cellular functions, our results highlight the importance of prioritizing core regulatory factors in the design of epigenetic interventions.

**Supplementary Note 3: Chromatin constraints govern differential performance of epigenetic tools**

In contrast to *STAT5B*, expression activation of *STAT5A* was not achieved under mastitis conditions, resulting in a failure to rescue casein synthesis. These results imply that mastitis-induced alterations in chromatin structure constitute a fundamental barrier to effective epigenetic editing at the *STAT5A* locus. In particular, mastitis-associated virulence factors, including peptidoglycan (PGN) and lipoteichoic acid (LTA) (Eckel & Ametaj, 2016; Yu et al., 2010), trigger sustained inflammatory signaling that promotes histone deacetylation and DNA hypermethylation via host mechanisms such as *HDAC* activation and *DNMT* upregulation (Chen et al., 2019; Wu et al., 2020). These epigenetic changes likely lead to chromatin condensation and reduced accessibility at the *STAT5A* promoter, thereby impeding sgRNA binding and docking of dCas9-based editors. Additionally, pathogen-associated molecular patterns (PAMPs) may activate innate immune pathways that further compromise chromatin openness or sequester transcriptional co-activators. Histone acetyltransferase dCas9-P300 core enhances chromatin accessibility through targeted histone acetylation (Hilton et al., 2015), whereas dCas9-VPR tripartite activator recruits multiple transcription factors (VP64, p65, Rta) to drive robust gene expression (Chavez et al., 2015). Our data suggest that the effectiveness of these epigenetic tools may be influenced by cell-type-specific chromatin context. Specifically, dCas9-P300 appears particularly efficient in compacted chromatin regions, possibly due to its direct histone-modifying activity, which can loosen nucleosome packing and increase chromatin openness. In contrast, dCas9-VPR may function more effectively in already accessible chromatin regions, where its multi-factor recruitment strategy synergizes with pre-existing transcriptional machinery to drive gene transcription (Wu et al., 2023). Consequently, the inflammatory milieu likely altered the local chromatin environment or imposed signaling inhibition that prevented *STAT5A* from being effectively activated by the dCas9-VPR system. The restoration of casein production mediated by *STAT5B* activation illustrates the promise of epigenetic approaches in inflammatory settings. This effect occurs through the molecular mechanism whereby both *STAT5A* and *STAT5B*, whether forming homodimers or heterodimers, can bind the β-casein promoter to activate transcription (Liu et al., 1996). Therefore, our results highlight the importance of considering chromatin context for gRNA design in therapeutic selection of tools.

**Supplementary Note 4: Limitations of the mastitis cell model using heat-inactivated *S. aureus***

We acknowledge that our mastitis model using heat-inactivated *S. aureus* has inherent limitations. Heat-inactivated bacteria lack virulence factors, such as exotoxins, extracellular enzymes, and immune-evasion proteins, which live *S. aureus* actively deploys to damage host tissues and evade immune defenses (Tam & Torres, 2019). As a result, our mastitis cell model primarily reflects host responses to bacterial structural components (e.g., peptidoglycan and lipoteichoic acid) rather than the complex interactions characteristic of live infection (Tsugami et al., 2021). Therefore, utilizing heat-inactivated bacteria fails to fully replicate the dynamic host–pathogen interactions characteristic of live infections, potentially simplifying the innate immune response and overlooking critical virulence mechanisms. Additionally, the observed acute cytokine peaks, including *IL-6*, *IL-8*, *IL-1β*, resemble a rapid response to pathogen-associated molecular patterns, which may differ from the low-grade, persistent inflammation characteristic of subclinical mastitis. Indeed, previous studies also suggests that live and heat-inactivated *S. aureus* can elicit distinct signaling pathways and cytokine profiles, further supporting that our model does not fully capture all aspects of *in vivo* infection (Liu et al., 2021; Zhang et al., 2023). Despite these limitations, our approach demonstrates that targeted epigenetic editing can restore lactation function, rather than provide broad-spectrum anti-inflammatory effects, following inflammatory challenge. While our approach does not yet directly translate into therapies against live bacterial infection, it establishes a proof-of-concept for lactation-focused interventions and offer a foundation for future studies in more physiologically relevant models. Importantly, this approach highlights a novel epigenetic strategy to restore milk nutrient components under inflammatory stress, which addresses a critical and underexplored aspect of mastitis management.

# SUPPLEMENTARY FIGURES

**Fig. S1. mRNA expression level of most lactogenic genes: signaling pathways (*AKT1*,** ***mTOR*, *PER2*, *PPrPγ*), lipid synthesis (*****FASN*, *SREBF1*), and protein synthesis (*****STAT5A*, *STAT5B*, *CSN2*, *JAK2*) in MAC-Ts.** *Ps* < 0.05, Student ’s t-test.


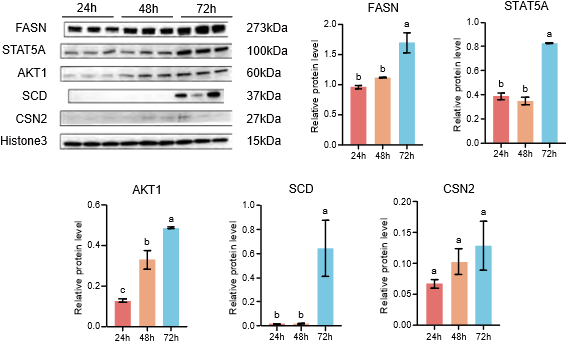


**Fig. S2.** **Western blots and their quantifications of FASN, PER2, STAT5A, AKT1, SCD, CSN2 in different groups (prolactin treatment for 24, 48, and 72 h) of MAC-T cells. Histone H3 was used as an endogenous control (n = 3 biological replicates).** *Ps* < 0.05, One-way ANOVA


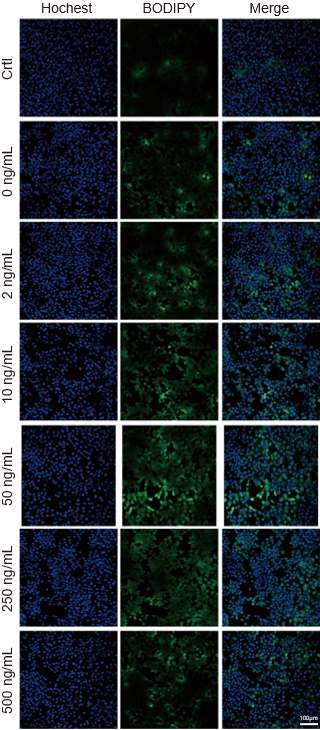

**Fig. S3. Fluorescence staining and quantification of BMECs treated with different concentration gradients of PRL for 72 h.** (n = 3) Above: BODIPY stains the lipid droplets, and Hoechst stains the cell nuclei; Below: The relative fluorescence intensity of lipid droplets as calculated by ImageJ (BODIPY/Hoechst). Blue, Hoechst; Green, BODIPY. Scale bar, 100μm. Crtl: No hormone treatment; 0–500 ng/mL: Experimental groups supplemented with varying concentrations of prolactin, under constant concentrations of insulin, hydrocortisone, and epidermal growth factor. *P* < 0.001, One-way ANOVA.


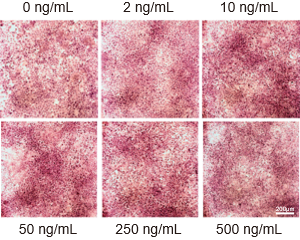


**Fig. S4. Oil Red O staining and quantification of BMECs treated with different concentration gradients of PRL for 72 h.** (n = 3) Above: Oil Red O stains the lipid droplets, and Hematoxylin stains the cell nuclei; Below: Quantitative analysis of 510nm absorbance after oil red staining. Dark blue, Hematoxylin; Red, Oil Red O. Scale bar, 200μm. Crtl: No hormone treatment; 0–500 ng/mL: Experimental groups supplemented with varying concentrations of prolactin, under constant concentrations of insulin, hydrocortisone, and epidermal growth factor. *P* < 0.001, One-way ANOVA.

**Fig. S5. Casein contents of BMECs treated with different concentration gradients of PRL** **for 72 h.** (n = 4) Crtl: No hormone treatment; 0–500 ng/mL: Experimental groups supplemented with varying concentrations of prolactin, under constant concentrations of insulin, hydrocortisone, and epidermal growth factor. *P* < 0.01, One-way ANOVA.

**Fig. S6. Triglyceride (TG) contents of BMECs treated with different concentration gradients of PRL** **for 72 h.** (n = 4) Crtl: No hormone treatment; 0–500 ng/mL: Experimental groups supplemented with varying concentrations of prolactin, under constant concentrations of insulin, hydrocortisone, and epidermal growth factor. *P* < 0.001, One-way ANOVA.

**Fig. S7.** **mRNA expression level of lactation-related genes signaling pathways (*AKT1*, *mTOR*, *PER2*), lipid synthesis (*FASN*, *ACACA*, *SCD*), and protein synthesis (*STAT5A*, *STAT5B*, *CSN2*, *JAK2*) in BMECs.** (n = 4) Crtl: No hormone treatment; 0–500 ng/mL: Experimental groups supplemented with varying concentrations of prolactin, under constant concentrations of insulin, hydrocortisone, and epidermal growth factor. *Ps* < 0.05, One-way ANOVA.


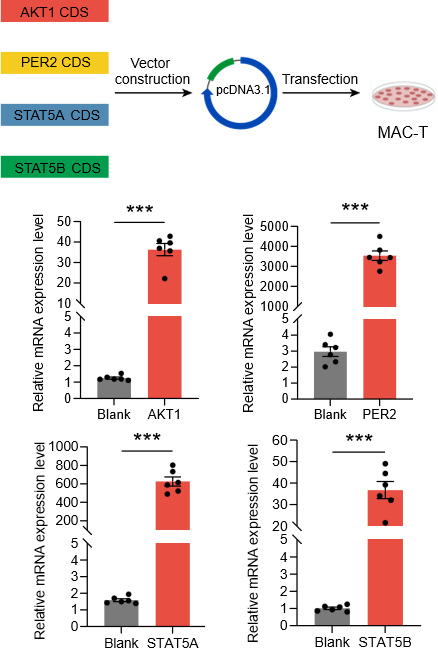


**Fig. S8. *AKT1*, *PER2*, *STAT5A* and *STAT5B* cDNA were cloned into the pcDNA3.1 vector for overexpression. The empty vector served as the control. q-PCR of overexpressing the *AKT1*, *PER2*, *STAT5A*, and *STAT5B* gene in MAC-T cells, *β-actin* served as control.** (n = 5) *P* < 0.0001, Student ’s t-test.


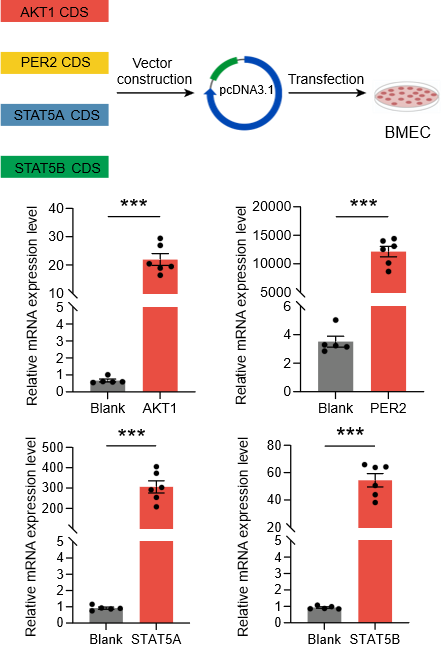


**Fig. S9. q-PCR of overexpressing the *AKT1*, *PER2*, *STAT5A*, and *STAT5B* gene in BMECs. β-actin was used as a control.** (n = 5) *P* < 0.0001, Student ’s t-test.


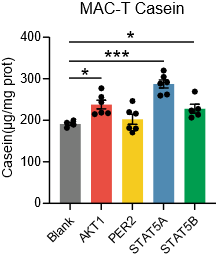


**Fig. S10. Casein content of overexpressing *AKT1*, *PER2*, *STAT5A*, and *STAT5B* gene in MAC-T cells.** (n = 5) *P* < 0.05, Student ’s t-test.


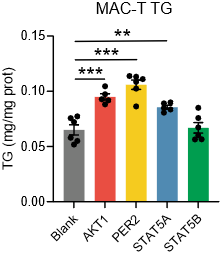


**Fig. S11. TG content of overexpressing *AKT1*, *PER2*, *STAT5A*, and *STAT5B* gene in MAC-T cells.** (n = 5) *P* < 0.05, Student ’s t-test.


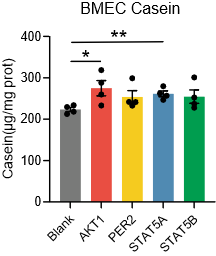


**Fig. S12. Casein content of overexpressing *AKT1*, *PER2*, *STAT5A*, and *STAT5B* gene in BMECs.** (n = 4) *P* < 0.05, Student ’s t-test.


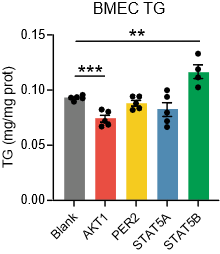


**Fig. S13. TG content of overexpressing *AKT1*, *PER2*, *STAT5A*, and *STAT5B* gene in BMECs.** (n = 4) *P* < 0.05, Student ’s t-test.

**
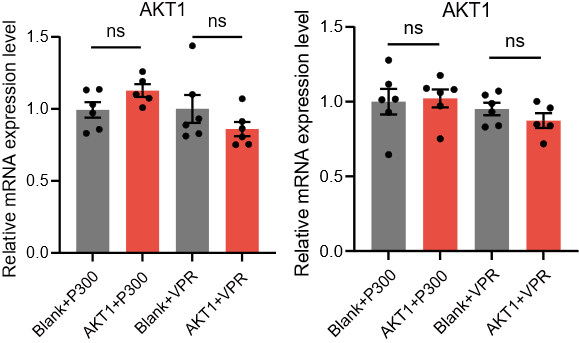
**

**Fig. S14. q-PCR results of the CRISPR activation screening of *AKT1* in MAC-T cells and BMECs.** (n = 5) Left: MAC-T cells; Right: BMECs. *P* > 0.05, Student ’s t-test.


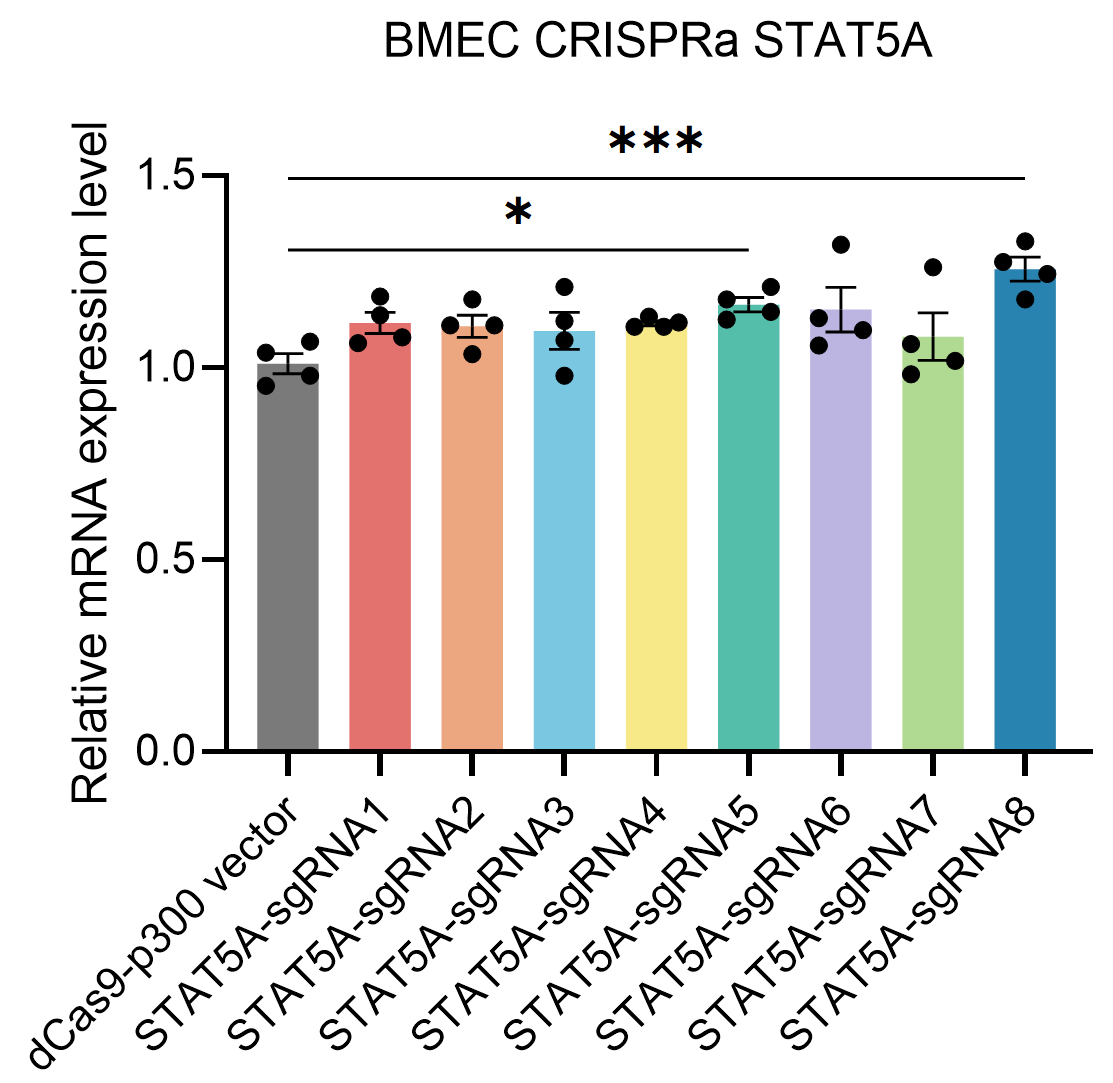


**Fig. S15.** **q-PCR results of single sgRNA-dCas9-P300 activation of *STAT5A* in BMECs, non-target sgRNA served as control.** (n = 4) * *P* < 0.05; *** *P* < 0.001, Student ’s t-test.

**
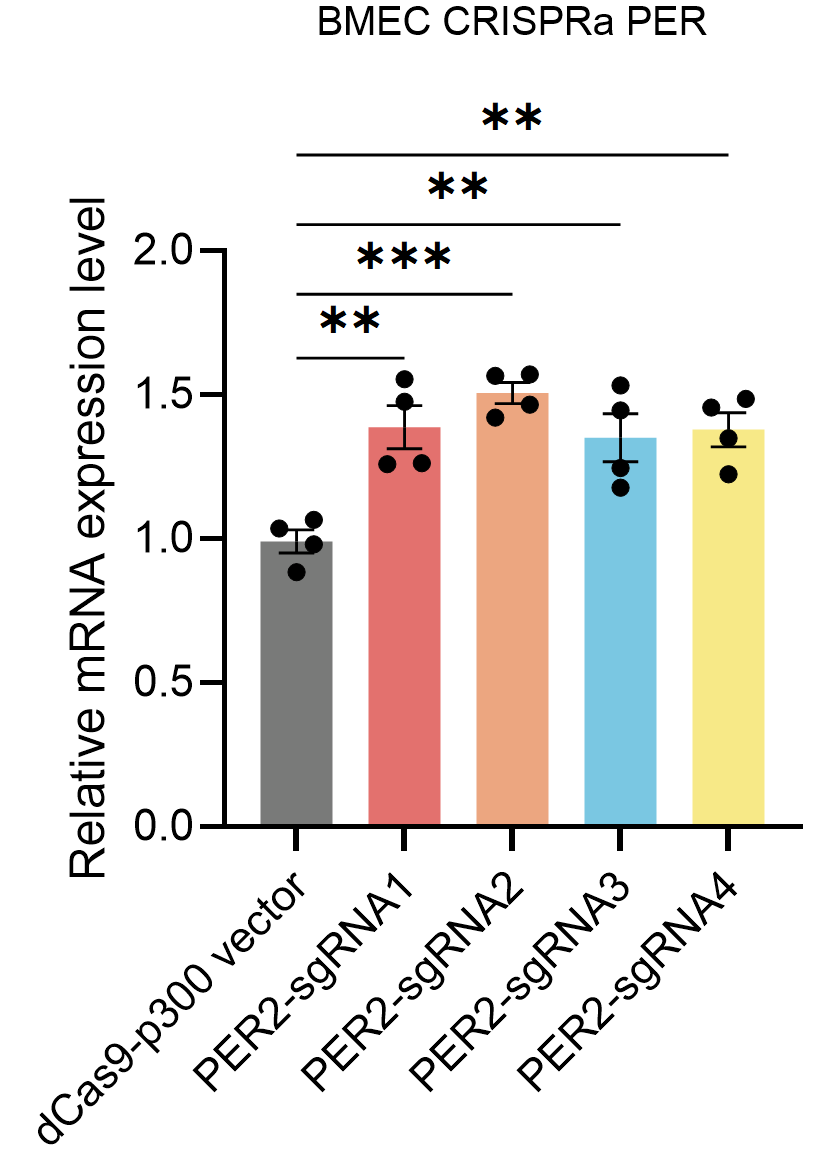
**

**Fig. S16. q-PCR results of single sgRNA-dCas9-P300 activation of *PER2* in BMECs, non-target sgRNA served as control.** (n = 4) ** *P* < 0.01; *** *P* < 0.001, Student ’s t-test.

**
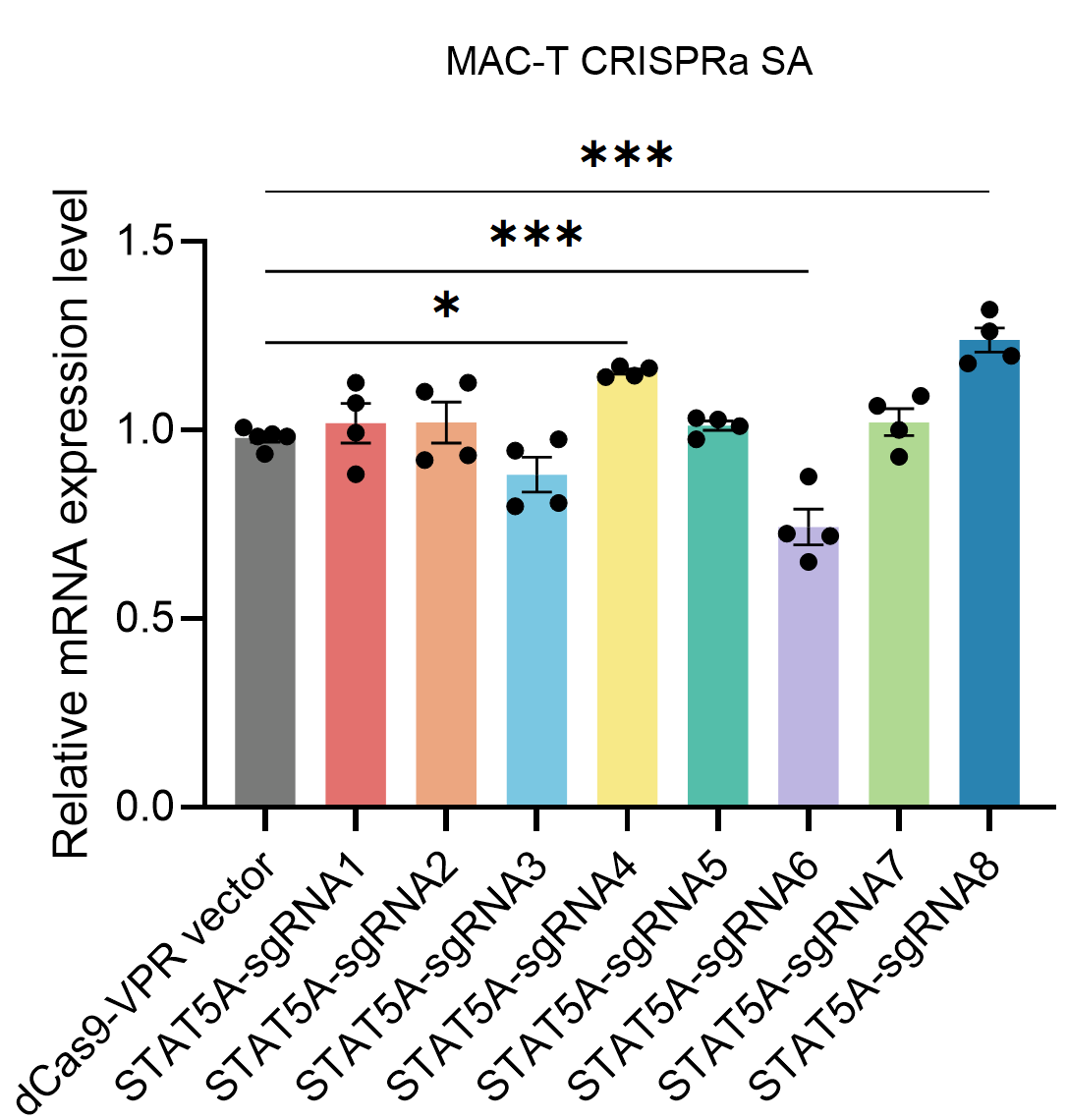
**

**Fig. S17. q-PCR results of single sgRNA-dCas9-VPR activation of *STAT5A* in MAC-Ts, non-target sgRNA served as control.** (n = 4) * *P* < 0.05; *** *P* < 0.001, Student ’s t-test.

**Fig. S18. q-PCR results of single sgRNA-dCas9-VPR activation of *PER2* in MAC-Ts, non-target sgRNA served as control.** (n = 4) * *P* < 0.05; ** *P* < 0.01, Student ’s t-test.

**
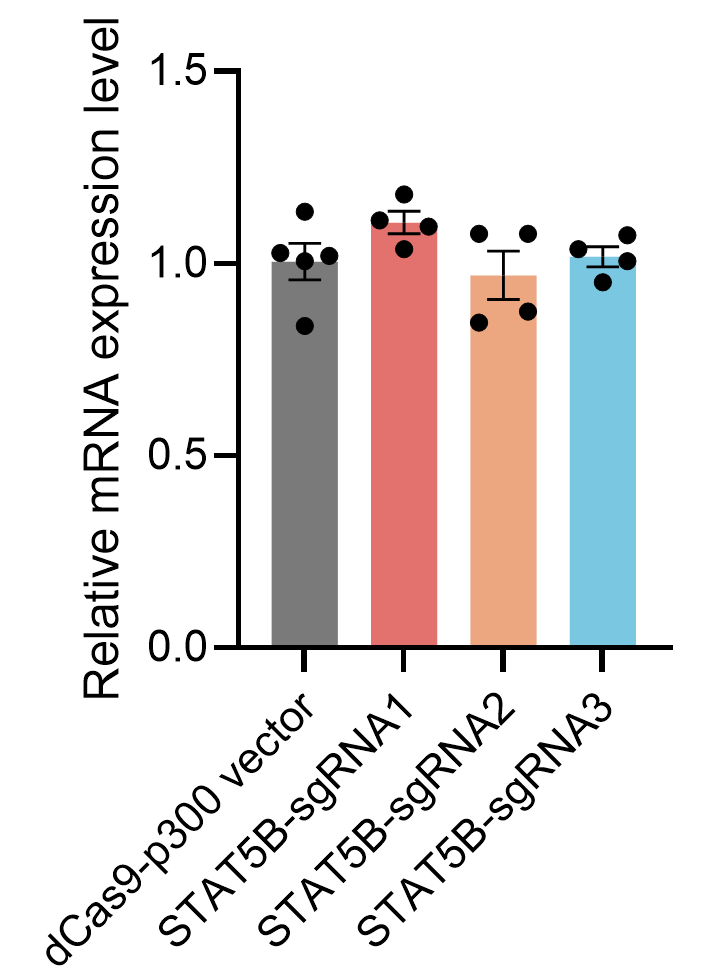
**

**Fig. S19. q-PCR results of single sgRNA-dCas9-P300 activation of *STAT5B* in MAC-Ts, non-target sgRNA served as control.** (n = 4) *Ps* > 0.05, Student ’s t-test.


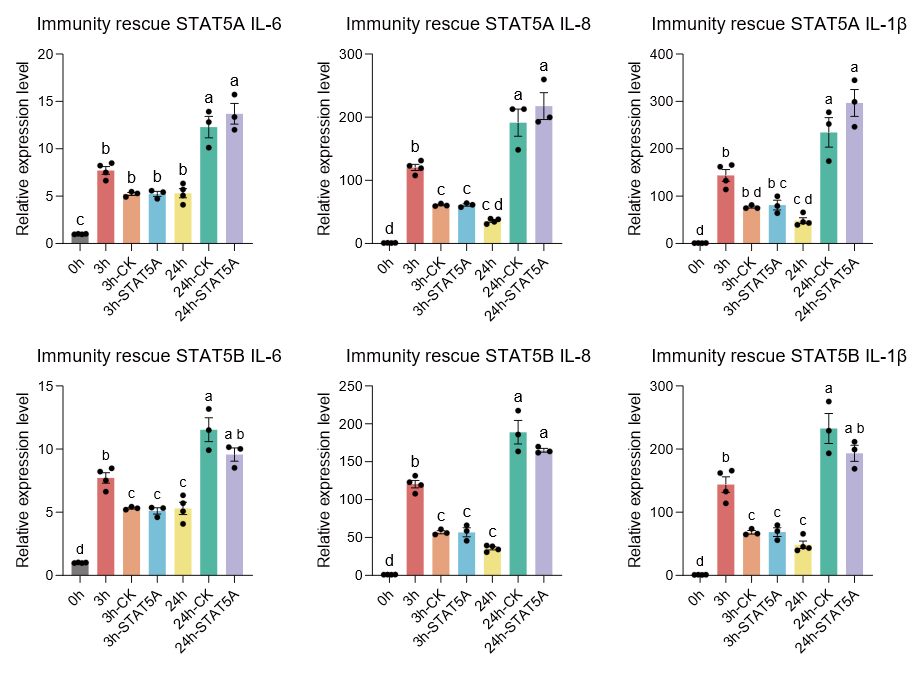


**Fig. S20. q-PCR results of *IL-6*, *IL-8* and *IL-1β* in the two types of cells after inflammatory state and epigenetic activation treatment.** (n = 3) *Ps* < 0.001, One-way ANOVA.


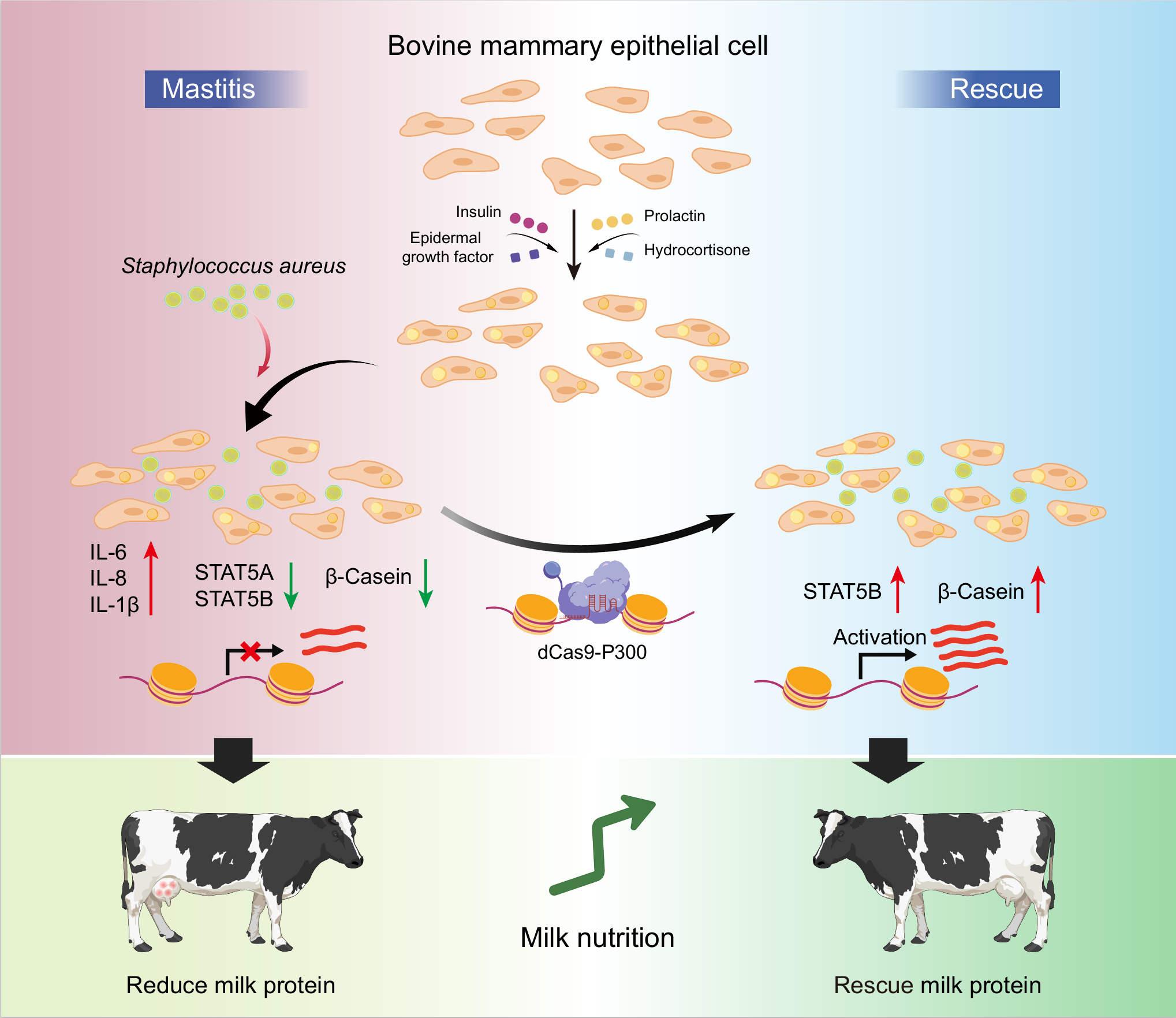


**Fig. S21. A model illustrating the mechanisms by which epigenetic editing restore nutrient component in the mastitis cell model.**


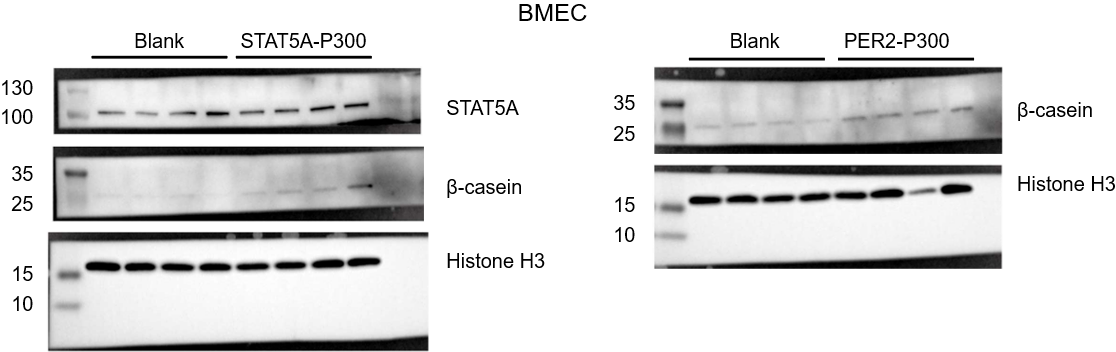


**Fig. S22. The original WB image of Figure 1G, and four replicates for both the Blank control and the STAT5A-P300 groups.**


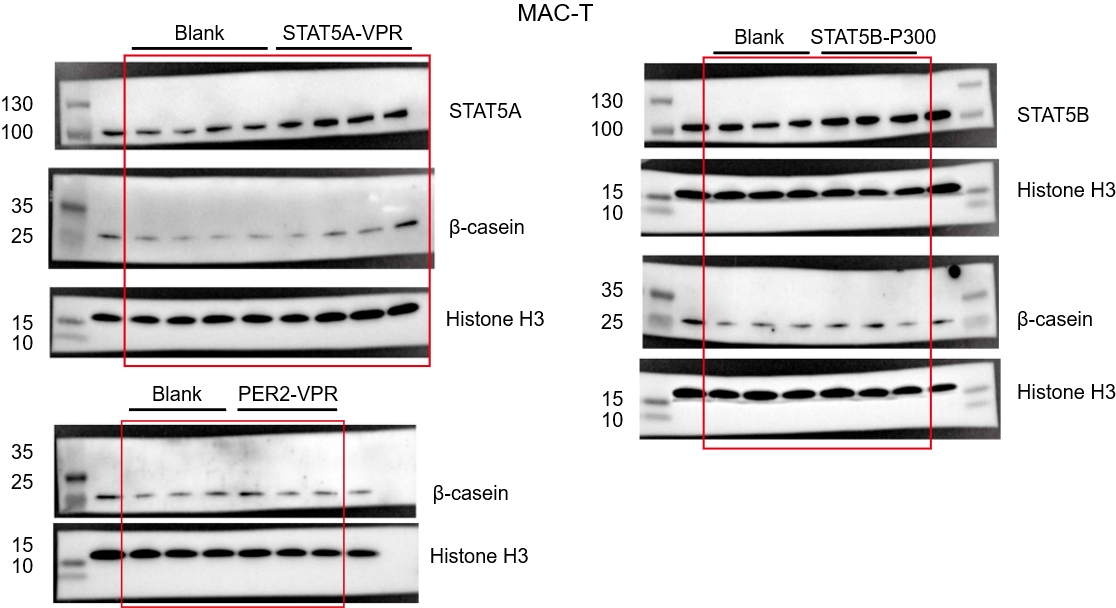


**Fig. S23. The uncropped western blot images for Figure 1J include data from four replicates for the Blank control, STAT5A-VPR, and PER2-VPR groups, and three replicates for the blank control and STAT5A-P300 groups.**


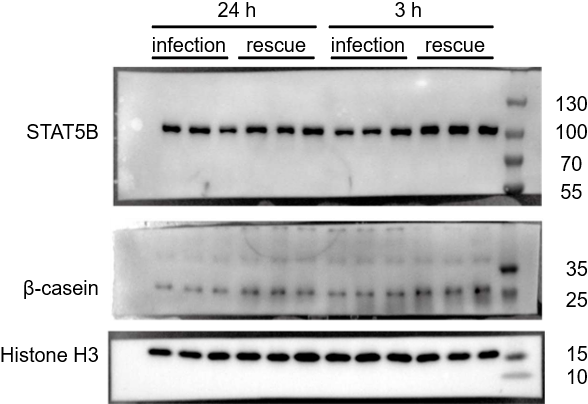


**Fig. S24. The original WB image of Figure 2P, and each group has three replicates.**

**Appendix Table S1 sgRNA sequence for the target genes**

| sgRNA | sequence (5’-3’) |
| --- | --- |
| STAT5A-gRNA1  STAT5A-gRNA2  STAT5A-gRNA3  STAT5A-gRNA4  STAT5A-gRNA5  STAT5A-gRNA6  STAT5A-gRNA7  STAT5A-gRNA8  STAT5B-gRNA1  STAT5B-gRNA2  STAT5B-gRNA3  PER2-gRNA1  PER2-gRNA2  PER2-gRNA3  PER2-gRNA4 | GAAGGAGAGAGCAACGCAGA  CCCGCAAGGCCTGTAGGCAG  GAGAGCGACCGAGGCTGGGA  GAGGAGGAAATCGCTGCTCT  CCGGACCGCCCGGCACGACC  AGCGGTGGCCGAGCCGTCCA  CACCGTCTTTCTTCCCGCAC  AGTCCACGCCTGTGACGAGA  GAGGCAGCTGACCTTTTGGA  CTTCTTCCTGTAACAGTGCA  TCTGCCGCTATCCTGGCTGC  CCGCCGCCAATGGCGGGCGC  TGACAGCGGCGAGTCCGCGC  GCTTTCGGCCGCAGCGGCGG  GCTTTCGGCCGCAGCGGCGG |

**Appendix** **Table S2 q-PCR Primers for the target genes**

| Gene | Primer sequence |
| --- | --- |
| MAC-T β-actin-F  MAC-T β-actin-R  BMEC β-actin-F  BMEC β-actin-R  AKT1-F  AKT1-R  mTOR-F  mTOR-R  β-casein-F  β-casein-R  Stat5A-F  Stat5A-R  PPARγ-F  PPARγ-R  FASN-F  FASN-R  Stat5B-F  Stat5B-R  ACACA-F  ACACA-R  SCD-F  SCD-R  SREBF1-F  SREBF1-R  JAK-2 F  JAK-2 R  PER2-F  PER2-R  IL‐6-F  IL‐6-R  IL‐8-F  IL‐8-R  IL-1β-F  IL-1β-R | TCAGCAAGCAGGAGTACGATGA  ATCCTGAGTCAAGCGCCAAA  AAGGACCTCTACGCCAACACG  TTTGCGGTGGACGATGGAG  TGGAGAACCTCATGCTGGACA  GCCGCACATCATCTCGTACAT  ATGCTGTCCCTGGTCCTTATG  GGTCAGAGAGTGGCCTTCAA  CTGGACCAGAGCCAGAGGAA  GAAAGCCAGAGCCTGACTCTCA  CCCTTCCCGTGGTTGT  ATGCCGTTGTAGTCCTC  GATAGGTGTGATCTTAACTGTCGGAT  CGCTAACAGCTTCTCCTTCTCG  CTGGAGCGTGAGCACAACCTG  GTGTGGAGTCCGTCAGCTCAT  CAGTCCTGGTGTGAGAAGTTGGC  TGATGTCAGTGATGGTGGCGTTG  AAGACGGACAAGCAGACGTT  TCCACTTCCAAAAAGAACTCAGAGA  CATGGCGTTCCAGAATGACG  AAGAAAAAGCCACGTCGGGA  ACGCCATCGAGAAACGCTAC  GTGCGCAGACTCAGGTTCTC  GAGCCATTCCCATGCAGAGTC  AGGCACCGGTTTCAGAAG  AGCGTGTTCCATAGCTCCAC  ATCTCGCTCTCGTGGCTTT  GCCTTCACTCCATTCGCTGTCTC  AAGTAGTCTGCCTGGGGTGGTG  AAGCTGGCTGTTGCTCTCTTGG  TTTGGGGTGGAAAGGTGTGGAAT  ATGACTTCCAAGCTGGCTGTTG  TTGATAAATTTGGGGTGGAAAG |

# References

Chavez, A., Scheiman, J., Vora, S., Pruitt, B. W., Tuttle, M., Iyer, E. P. R., Lin, S. L., Kiani, S., Guzman, C. D., Wiegand, D. J., Ter-Ovanesyan, D., Braff, J. L., Davidsohn, N., Housden, B. E., Perrimon, N., Weiss, R., Aach, J., Collins, J. J., & Church, G. M. (2015). Highly efficient Cas9-mediated transcriptional programming. *Nature Methods*, *12*(4), 326-U365. <https://doi.org/10.1038/Nmeth.3312>

Chen, J. B., Wu, Y. J., Sun, Y. W., Dong, X. W., Wang, Z. L., Zhang, Z., Xiao, Y. L., & Dong, G. Z. (2019). Bacterial endotoxin decreased histone H3 acetylation of bovine mammary epithelial cells and the adverse effect was suppressed by sodium butyrate. *Bmc Veterinary Research*, *15*. <https://doi.org/10.1186/s12917-019-2007-5>

Doench, J. G., Fusi, N., Sullender, M., Hegde, M., Vaimberg, E. W., Donovan, K. F., Smith, I., Tothova, Z., Wilen, C., Orchard, R., Virgin, H. W., Listgarten, J., & Root, D. E. (2016). Optimized sgRNA design to maximize activity and minimize off-target effects of CRISPR-Cas9. *Nature Biotechnology*, *34*(2), 184-+. <https://doi.org/10.1038/nbt.3437>

Eckel, E. F., & Ametaj, B. N. (2016). Role of bacterial endotoxins in the etiopathogenesis of periparturient diseases of transition dairy cows. *Journal of Dairy Science*, *99*(8), 5967-5990. <https://doi.org/10.3168/jds.2015-10727>

Hilton, I. B., D'Ippolito, A. M., Vockley, C. M., Thakore, P. I., Crawford, G. E., Reddy, T. E., & Gersbach, C. A. (2015). Epigenome editing by a CRISPR-Cas9-based acetyltransferase activates genes from promoters and enhancers. *Nature Biotechnology*, *33*(5), 510-U225. <https://doi.org/10.1038/nbt.3199>

Jaeger, A., Bardehle, D., Oster, M., Günther, J., Muráni, E., Ponsuksili, S., Wimmers, K., & Kemper, N. (2015). Gene expression profiling of porcine mammary epithelial cells after challenge with and in vitro. *Veterinary Research*, *46*(1), 50. <https://doi.org/10.1186/s13567-015-0178-z>

Liu, J., Wei, X. F., Zhang, Y., Ran, Y. X., Qu, B., Wang, C. M., Zhao, F., & Zhang, L. (2024). dCas9-guided demethylation of the AKT1 promoter improves milk protein synthesis in a bovine mastitis mammary gland epithelial model induced by using. *Cell Biology International*, *48*(3), 300-310. <https://doi.org/10.1002/cbin.12106>

Liu, K., Mao, W., Liu, B., Li, T. T., Wu, J. D., Fu, C. Q., Shen, Y., Pei, L., & Cao, J. S. (2021). Live S. aureus and heat-killed S. aureus induce different inflammation-associated factors in bovine endometrial tissue in vitro. *Molecular Immunology*, *139*, 123-130. <https://doi.org/10.1016/j.molimm.2021.07.015>

Liu, X. W., Robinson, G. W., & Hennighausen, L. (1996). Activation of Stat5a and Stat5b by tyrosine phosphorylation is tightly linked to mammary gland differentiation. *Molecular Endocrinology*, *10*(12), 1496-1506. <https://doi.org/10.1210/me.10.12.1496>

Ran, F. A., Hsu, P. D., Wright, J., Agarwala, V., Scott, D. A., & Zhang, F. (2013). Genome engineering using the CRISPR-Cas9 system. *Nature Protocols*, *8*(11), 2281-2308. <https://doi.org/10.1038/nprot.2013.143>

Sajjanar, B., Trakooljul, N., Wimmers, K., & Ponsuksili, S. (2019). DNA methylation analysis of porcine mammary epithelial cells reveals differentially methylated loci associated with immune response against

challenge. *BMC Genomics*, *20*(1), 623. <https://doi.org/10.1186/s12864-019-5976-7>

Tam, K., & Torres, V. J. (2019). Secreted Toxins and Extracellular Enzymes. *Microbiology Spectrum*, *7*(2). <https://doi.org/10.1128/microbiolspec.GPP3-0039-2018>

Tsugami, Y., Wakasa, H., Kawahara, M., Nishimura, T., & Kobayashi, K. (2021). Lipopolysaccharide and lipoteichoic acid influence milk production ability via different early responses in bovine mammary epithelial cells. *Experimental Cell Research*, *400*(2), 112472. <https://doi.org/10.1016/j.yexcr.2021.112472>

Wu, Q. X., Wu, J. J., Karim, K., Chen, X., Wang, T. Y., Iwama, S., Carobbio, S., Keen, P., Vidal-Puig, A., Kotter, M. R., & Bassett, A. (2023). Massively parallel characterization of CRISPR activator efficacy in human induced pluripotent stem cells and neurons. *Molecular Cell*, *83*(7), 1125-+. <https://doi.org/10.1016/j.molcel.2023.02.011>

Wu, Y. J., Chen, J. B., Sun, Y. W., Dong, X. W., Wang, Z. L., Chen, J. C., & Dong, G. Z. (2020). PGN and LTA from Induced Inflammation and Decreased Lactation through Regulating DNA Methylation and Histone H3 Acetylation in Bovine Mammary Epithelial Cells. *Toxins*, *12*(4). <https://doi.org/10.3390/toxins12040238>

Yu, C., Shi, Z. R., Chu, C. Y., Lee, K. H., Zhao, X., & Lee, J. W. (2010). Expression of bovine granulocyte chemotactic protein-2 (GCP-2) in neutrophils and a mammary epithelial cell line (MAC-T) in response to various bacterial cell wall components. *Veterinary Journal*, *186*(1), 89-95. <https://doi.org/10.1016/j.tvjl.2009.07.012>

Zhang, K., Jia, Y., Qian, Y., Jiang, X., Zhang, S., Liu, B., Cao, J., Song, Y., & Mao, W. (2023). Staphylococcus aureus increases Prostaglandin E2 secretion in cow neutrophils by activating TLR2, TLR4, and NLRP3 inflammasome signaling pathways. *Frontiers in Microbiology*, *14*. <https://doi.org/10.3389/fmicb.2023.1163261>
